# COVID-19 vaccinology landscape in Africa

**DOI:** 10.3389/fimmu.2022.955168

**Published:** 2022-12-05

**Authors:** Sara Baptista, Sanushka Naidoo, Sara Suliman, Emmanuel Nepolo, Bernard N. Kanoi, Jesse Gitaka, Oyedemi Mbaebie Blessing, Shymaa Enany

**Affiliations:** ^1^ Department of Biology & Physiology of Malaria, Next Einstein Forum Community of Scientists, Kigali, Rwanda; ^2^ Instituto de Medicina Molecular, João Lobo Antunes, Faculdade de Medicina, Universidade de Lisboa, Lisboa, Portugal; ^3^ Department of Biochemistry, Genetics and Microbiology, Forestry and Agricultural Biotechnology Institute (FABI), University of Pretoria, Pretoria, South Africa; ^4^ Zuckerberg San Francisco General Hospital, Division of Experimental Medicine, University of California San Francisco, San Francisco, CA, United States; ^5^ Chan Zuckerberg Biohub, San Francisco, CA, United States; ^6^ Department of Human, Biological and Translational Medical Sciences, School of Medicine, University of Namibia, Windhoek, Namibia; ^7^ Centre for Research in Infectious Diseases, Directorate of Research and Innovation, Mount Kenya University, Thika, Kenya; ^8^ Department of Biotechnology, Michael Okpara University of Agriculture, Umudike, Nigeria; ^9^ Department of Microbiology and Immunology, Faculty of Pharmacy, Suez Canal University, Ismailia, Egypt

**Keywords:** vaccinology, Africa, COVID - 19, local manufacture, improved access, vaccines

## Abstract

More than two years after the start of COVID-19 pandemic, Africa still lags behind in terms vaccine distribution. This highlights the predicament of Africa in terms of vaccine development, deployment, and sustainability, not only for COVID-19, but for other major infectious diseases that plague the continent. This opinion discusses the challenges Africa faces in its race to vaccinate its people, and offers recommendations on the way forward. Specifically, to get out of the ongoing vaccine shortage trap, Africa needs to diversify investment not only to COVID-19 but also other diseases that burden the population. The continent needs to increase its capacity to acquire vaccines more equitably, improve access to technologies to enable local manufacture of vaccines, increase awareness on vaccines both in rural and urban areas to significantly reduce disease incidence of COVID-19 and as well as other prevalent diseases on the African continent such as HIV and TB. Such efforts will go a long way to reduce the disease burden in Africa.

## Introduction

Development of vaccines to prevent COVID-19 diseases occurred with unprecedented speed. These vaccines, (Pfizer-BioNTech, Moderna, Johnson & Johnson Janssen, AstraZeneca, Novavax COVID-19, Sputnik and Sinopharm, and others) with different modes of action, efficacy, and potential side effects, are still not available to several communities in various countries including those on the African continent, that face additional disease burden. According to WHO African region, a month before the milestone set for all countries to vaccinate 70% of their people against COVID-19, a total of 169 million people have been fully vaccinated (1). This represents a mere 14.7% of the eligible individuals. Only two countries, Seychelles and Mauritius, have achieved the set target with 12 countries still lagging below 10%. This is, in part, due to unavailability of the needed vaccines since only 40% (56 doses per 100 population) of the doses required to reach 70% of people have been received in all countries. In addition, of the received doses, countries have only administered 55% with 26/46 (59%) countries administering fewer than 50% of doses received (1). Moreover, there are high rates of COVID-19 vaccine hesitancy especially in Western and Central Africa (2). All these factors have highlighted the critical challenge Africa faces in terms of vaccine development, availability, deployment, and sustainability, not only for COVID-19, but for other major infectious diseases that plague the continent. This gap needs to be addressed.

It’s worthwhile to note that there have been success stories with immunizations programs in the African continent over the past decades. The Expanded Programme on Immunization (EPI) efforts drastically reduced child mortality caused by several vaccine-preventable diseases worldwide, raising immunization coverage from 5% in 1970s to more than 80% in 1990s and globally preventing around 2.5 million deaths every year (3). This was mainly driven by strategies that helped strengthen leadership, management competencies and logistical support that overcome weaknesses in the primary health care systems thus ensuring continuity of services in light of low education, financial challenges, and conflicts (4). Indeed, several global initiatives emerged to develop and distribute vaccines, particularly to low- and middle-income countries (LMICs). Measles decreased by 92% in Africa due to efforts of the World Health Organization (WHO) partnership with United Nations Children’s Fund between 2001 and 2009 (3). The Global Alliance for Vaccines and Immunization (Gavi), set up in 2000, facilitated the vaccination of more than 822 million children between 2000 and 2019, preventing more than 14 million deaths in LMICs (5). In 2012, 194 Member States of the World Health Assembly endorsed the Global Vaccine Action Plan (GVAP), which targeted around 90 LMICs, with the introduction of several new vaccines in Africa (6).

**Figure 1:**
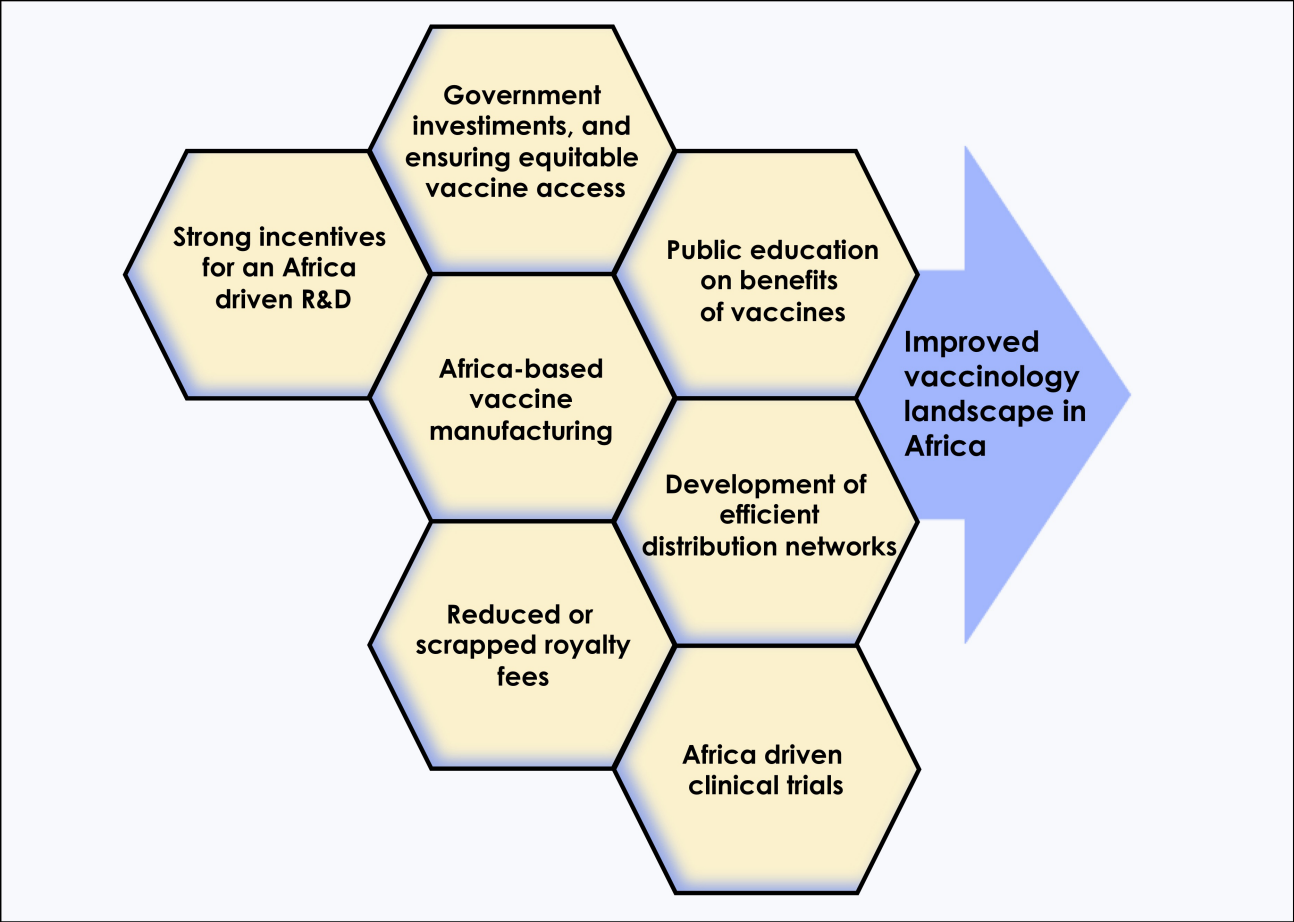
In order to alter the current COVID-19 vaccinology landscape in Africa, multiple approaches such as increased investments by African governments to support vaccines research and development, improved access to technologies that boost local manufacturing, and public education on benefits of vaccines, among others, will be needed.

Under this paradigm, why couldn’t African countries autonomously enhance their local vaccines manufacturing and commercialization capacity to an important source of income, similar to what happened with other LMICs, such as Cuba, Brazil and India? Despite several significant achievements, the global weight of infectious diseases remains disproportionately heavier in Africa compared to other continents. Incomplete, invalid, unequal, and heterogenous immunization coverage across the continent, and within countries, are still the major contributors to child mortality and morbidity in Africa. In 2020, the WHO launched the COVAX initiative, a vaccine access component of the Access to COVID-19 Tools (ACT) accelerator to ensure equitable global access to COVID-19 vaccines. However, with pressing demand for vaccine access in the United States, Europe and other high income countries, which bought more than their needed vaccine supplies, marketing incentive to prioritize deployment of these vaccines in and for LMICs substantially decreased. The same situation happened in Central and West Asia (7) which in turn pushed the public health policy experts to demand a greater need for global solidarity in vaccine access (8). We believe that one solution to decisively and irreversibly address this gap is to shift the development and production of vaccines for Africa in Africa (**Figure 1**).

## Africa investment in vaccines

Implementation of immunization programs in Africa suffers four main obstacles: economic and political, medical and scientific, structural and demographic, and societal and cultural factors (9). This very challenges have hindered governments and private entities to invest in vaccines production. Lack of political will and absence of clarity on the immediate economic benefits has a times hindered the sitting governments to support vaccine and pharmaceutical programs. Many African countries lack strong and effective disease surveillance capabilities driven majorly by weak scientific training base. In addition, societal and cultural issues, obstacles relate to poverty, illiteracy (especially among women), religious taboos, superstition, and an overemphasis on curative rather than preventive medicine (9). These factors would normally lead to lack of awareness of the strong economic importance of vaccination, thus reduced interests in governments budgetary allocation and lack of motivation to private entities.

WHO Global spending on health report showed that health expenses continually rose between 2000 and 2018 and reached US$ 8.3 trillion or 10% of global GDP. However, Africa health care expenses remained lower than the global average at US$38 per capita, about 4.4% of GDP with governments spending US$9 per capita in 2018, about 1.2% of GDP (10, 11). The situation is exacerbated by government debts for example, in sub-Saharan region, where both domestic and internal debt either remained stable or marginally increased since 2017 (11). The health care sector had a financing gap of US$ 66 billion in 2019, with governments dedicating only a small percentage of their national budget to routine immunization programs (12). This implies that sustainability of vaccines and immunization programs in African countries remains a challenge. Of the initial 75-eligible Gavi countries, in 2016, only four countries started full self-financing of all vaccines and with Gavi support (13). Thus, although more than US$1 billion had been paid by countries in co-financing contributions since Gavi’s policy was introduced, only 1 (Angola) of the 16 countries had transitioned out of Gavi’s support by 2019. Adding to that, under these circumstances, forecasting vaccine development and demand in African countries is crucial for forecasting sustainability, cost control, and socio-economic development since less than 1% of vaccines used in Africa are made in Africa, leaving African countries vulnerable to future pandemics akin to COVID-19.

The effect COVID-19 on the control of neglected tropical diseases (NTD), such as hookworm in school-aged children, schistosomiasis, and onchocerciasis cannot be underestimated. These NTDs have long term economic impact on the affected populations, such as through limiting educational opportunities for young children by interfering with cognitive development and causing undesirable effects on child development, thus trapping the affected individuals in a cycle of poverty (11, 14). Unfortunately, the most vulnerable population earn less than a dollar a day making NTD vaccines a less attractive business opportunity by pharmaceutical companies from high-income countries (11). This calls for African governments to consider investing in vaccine research, developing and production against the NTDs.

Concerning vaccines already developed for high-income countries, diversifying production to Africa could be a “win-win” situation. In this case: (i) LMICs get access to a product that would have been unattainable if vaccines were produced outside the continent, (ii) producers benefit from increased revenues and profits, and (iii) high income countries benefit from slightly lower prices due to reduced demand. Other option include vaccines intellectual property rights (IPRs) waiver. This is currently a key hinderance to the actualization of global vaccine access (15).

Nevertheless, there are many factors to consider prior to commitment in advancing Africa’s vaccinology environment. This include high cost and time required to establish complex processes, and capabilities for production of a broad portfolio of vaccines, fragmented or inconsistent demand, diverse regulatory requirements, and limited local competence and experience (16). Another important consideration, is how to strengthen clinical trials oversight in Africa and promote developed standardized protocols at the country and regional level, specially that Africa has a tremendous network of clinical trial sites for infectious diseases like TB, Malaria and HIV. Considering the complexity of introducing new vaccines and that such decisions relate to an important public health programme, it is crucial that these decisions are well-informed, unbiased and locally relevant, with maximized benefits on population-based evidence (17).

Clinical trials to license COVID-19 vaccines have not “cut corners” to expedite distribution, but have in fact been conducted in compliance with international regulatory standards prior to marketing. However, data should be collected from populations that are representative of those intended to use vaccines. Africa was suggested early in the pandemic to run trials quickly because of a knowledge that it would be easier there. However, Africa has not reaped the rewards of these trials with inequitable global vaccine distribution. In-depth interviews with experts in manufacturing capacity, implementation and regulatory capacity building in Africa, revealed attractiveness for the African vaccine manufacturing market (9). Although vaccine manufacturers from high-income countries may be interested in empowering African nations to develop their own manufacturing capacity, they are very reluctant to expand their capacities to Africa, with wealthy countries (particularly the European Union, the United Kingdom, Switzerland, and the United states) defending the Big Pharma and/or refusing to sign Trade-Related Aspects of Intellectual Property Rights (TRIPS) waivers (15). In contrast, African governments are interested in establishing vaccine manufacturing capacity in their countries but with an external financial despite existing technical capacity (9). Moreover, several international partnerships have strengthened regulatory oversight and sustainable clinical trial infrastructure, during clinical development of vaccines in developing countries (18).

In Africa, vaccine research and development, although unevenly distributed across the continent, is mainly financed by the public sector (65%), followed by philanthropic donors and private industry, which finance 21% and 14%, respectively (16). Almost all African countries are home to at least one large research institute, necessitating the need to link research to industry. Indeed, over the last few years, Africa has extended sites conducting human vaccine manufacturing from 2 to 6 sites distributed in 5 countries: Institute Pasteur de Dakar (in Senegal), Institute Pasteur de Tunis (in Tunisia), Biovaccines and Innovate Biotech (both in Nigeria), Vacsera (in Egypt) and Biovac Institute (in South Africa). With the coming of COVID-19, efforts are being made to increase the vaccine manufacturing capacity in Africa with at least twelves COVID-19 production facilities developed or earmarked across six African member states (19). Undoubtedly, the African vaccinology landscape must change. There is an urgent need for African governments to strengthen national investments in science education and research. Following and consistent with the Bamako call for research investment, African countries need to commit to invest a significant portion of health budgets to health research (20). However, many countries are yet met this goal.

The current medical research priorities remain undiversified (with Tuberculosis, HIV and Malaria receiving more than 80% of total funding) and unevenly distributed across the continent, and particularly limited in Central Africa region, which harbours the most populous countries and where risk of emerging pathogens is highest. Nevertheless, Africa should not jeopardize the ongoing large-scale and routine immunization campaigns to avoid reversal of decades of progress against other infectious diseases in the continent (21). Africa driven R&D initiates have an important role in the continent’s fight against endemic diseases and in the global fight against emerging outbreaks. The world has the responsibility to acknowledge and promote Africa’s equal inclusion in this mission.

## Africa vaccine accessibility and manufacturing capability

Equitable access to COVID-19 vaccines has been a major concern during pandemic, with many healthcare leaders warning that vaccine nationalism would allow the virus to continue thriving and lead to emerging variants of concern (22). There have been some small but positive developments in this area. For instance, in South Africa, a consortium was developed between the Medicines Patent Pool (MPP) and WHO, Afrigen Biologics (PTY) Limited, Biovac, the South African Medical Research Council (SAMRC) and Africa CDC, to address the inequality in vaccine manufacturing capability. The priority is to ensure technology transfer and to manufacture vaccines in Africa to respond more equitably to current and future pandemics.

## Vaccine hesitancy

Alongside increasing vaccine accessibility and manufacturing capability on the African continent, vaccine hesitancy, a major barrier to the successful adoption of immunization efforts, needs to be addressed (23). The social, demographic and education factors influencing vaccine hesitancy is not well defined however, the successful uptake of COVID-19 vaccines in some African countries, such as Botswana, demonstrates how coordinated education campaigns, distribution of vaccines to rural and urban settings, deployment of health-care workers and prioritizing vulnerable demographic groups, have mitigated the social barrier of vaccine hesitancy.

## Conclusion and recommendations

In conclusion, Africa needs; (i) to diversify investment in diseases that burden the population, (ii) an increase in its capacity to acquire vaccines more equitably, (iii) improved access to technologies to enable local manufacture of vaccines, (iv) an increase in vaccine education awareness campaigns and deployment at rural, urban and peri-urban areas to significantly reduce disease incidence of COVID-19 and other diseases that are prevalent on the African continent such as HIV and TB. Such efforts will go a long way to reduce the disease burden in Africa.

## Author contributions

All authors listed have made a substantial, direct, and intellectual contribution to the work and approved it for publication.

## Funding

SS was supported by an award from the Massachusetts Life Sciences Center Accelerating Coronavirus Testing Solutions (A.C.T.S), Nina Ireland Program for Lung Health, and the Chan Zuckerberg Biohub Initiative. JG and BNK were supported by Africa Academy of Sciences funding for Covid-19 Research & Development goals for Africa (SARSCoV2-4-20-010). The funding sources had no role in study design, collection, analysis, interpretation of data, and publication.

## Conflict of interest

The authors declare that the research was conducted in the absence of any commercial or financial relationships that could be construed as a potential conflict of interest.

## Publisher’s note

All claims expressed in this article are solely those of the authors and do not necessarily represent those of their affiliated organizations, or those of the publisher, the editors and the reviewers. Any product that may be evaluated in this article, or claim that may be made by its manufacturer, is not guaranteed or endorsed by the publisher.
